# O.1.1-1 Burnout and recovery need among Flemish secondary school teachers: what type of physical activity-intensity matters?

**DOI:** 10.1093/eurpub/ckad133.077

**Published:** 2023-09-11

**Authors:** Yanni Verhavert, Elke Van Hoof, Evert Zinzen, Kristine De Martelaer, Jelle Van Cauwenberg, Tom Deliens

**Affiliations:** Vrije Universiteit Brussel, Belgium; Vrije Universiteit Brussel, Belgium; Vrije Universiteit Brussel, Belgium; Vrije Universiteit Brussel, Belgium; Universiteit Gent, Belgium; yanni.verhavert@vub.be; Vrije Universiteit Brussel, Belgium

## Abstract

**Purpose:**

Teacher burnout is a topic of concern for educational institutions and thus it is important to investigate how teacher burnout can be managed or prevented. Previous research showed that physical activity (PA) may be effective in reducing burnout, but research regarding the optimal PA-intensity is lacking. Therefore, the aim of this study was to investigate the association between PA-intensities, burnout and recovery need in secondary school teachers in Flanders.

**Methods:**

One thousand nine hundred and nine secondary school teachers were included in this cross-sectional study. In September-October 2019, all teachers completed an online-questionnaire assessing burnout dimensions (i.e., emotional exhaustion, depersonalization and personal accomplishment), recovery need, and PA-intensities per domain (i.e., walking, moderate-intensity PA and vigorous-intensity PA in the following domains: work-related PA, active transport, leisure-time PA, domestic & garden PA), as well as sociodemographic and work-related factors. Multiple linear regression models were applied to identify which PA intensities are significantly associated with the burnout dimensions and recovery need. Adjusted (i.e., adjusted for all PA intensities within each PA domain) and unadjusted models (i.e., PA intensities per domain separately) were created.

**Results:**

The unadjusted models showed that depersonalization was positively associated with moderate-intensity PA at work and moderate-intensity domestic & garden PA, and that more personal accomplishment was associated with more walking at work and during transport. The adjusted models showed that emotional exhaustion and recovery need were negatively associated with vigorous-intensity PA during leisure-time and positively associated with moderate-intensity domestic & garden PA, and that more depersonalization was associated with more walking during leisure-time.

**Conclusion:**

Walking at work, during transport and during leisure-time, moderate-intensity PA at work, vigorous-intensity PA during leisure-time and moderate-intensity domestic & garden PA may be important factors when it comes to managing or preventing burnout and recovery need. Due to the cross-sectional study design, no conclusions about the causal relationship can be made. Future research should use longitudinal or experimental designs to get more insight into causality.

